# The multifaceted role of microbiota in liver cancer: pathogenesis, therapy, prognosis, and immunotherapy

**DOI:** 10.3389/fimmu.2025.1575963

**Published:** 2025-06-16

**Authors:** Yun Feng, Meng-Zhen Han, Yu-Hang Zhou, Yi-Wen Wang, Yue Wang, Tao Sun, Jun-Nan Xu

**Affiliations:** ^1^ Department of Breast Medicine 1, Cancer Hospital of China Medical University, Liaoning Cancer Hospital, Shenyang, China; ^2^ Department of Pharmacology, Cancer Hospital of China Medical University, Liaoning Cancer Hospital, Shenyang, China; ^3^ Department of Breast Medicine, Cancer Hospital of Dalian University of Technology, Liaoning Cancer Hospital, Shenyang, China

**Keywords:** carcinogenesis, hepatocellular carcinoma, microbiota, therapy, prognosis, immunotherapy

## Abstract

Accumulating evidence suggests that the progression of hepatocellular carcinoma (HCC) is intricately associated with dynamic alterations in microbiota composition. Disruption of gut microbial homeostasis enables pathogenic gut bacteria to translocate to the liver via the gut-liver axis, where they modulate the tumor microenvironment to promote HCC development. Also, they are associated with anti-tumor immune responses. Studies have confirmed that the microbiota exhibits potential as a biomarker for predicting immunotherapy responses, and its can improve clinical efficacy in the treatment of HCC.This review systematically evaluates current evidence elucidating the regulatory mechanisms by which the microbiota governs the progression of HCC, and explores its synergistic interactions with therapeutic strategies for HCC.

## 1 Introduction

Liver cancer is among the most prevalent cancers in China (1). Hepatocellular carcinoma (HCC) represents 80% of liver cancer (2). Chronic liver conditions mainly encompass non-alcoholic fatty liver disease (also known as steatosis), alcoholic liver disease (referred to as ALD), liver injury caused by medications, and viral hepatitis (3). Chronic liver diseases can cause continuous liver damage, and as the conditions worsen, it can lead to cirrhosis and potentially HCC (3). In this context, the microbiota has emerged as a central focus and a critical determinant implicated in HCC.

The microbiota including bacteria, fungi, and viruses (4). Under physiological conditions, the gut microbiota maintains a homeostatic equilibrium. However, compromised intestinal barrier function and alterations in the gut microenvironment drive the expansion of opportunistic pathogens such as *Escherichia coli* and Proteobacteria, resulting in dysbiosis and diseases. Researchers have identified the presence of hepatic and intratumoral microbiota through the application of high-throughput technologies (5). The existence of the gut-liver axis suggests that hepatic and intratumoral microbiota are potentially derived from the gut. The gut microbiota and hepatic microbiota play critical roles in the pathogenesis, therapeutic and prognosis modulation of HCC. Therefore, microbiota-modulating strategies (e.g., probiotics, fecal microbiota transplantation [FMT], and antibiotics) exhibit therapeutic potential in HCC by reshaping microbial composition.

HCC, a high-incidence malignancy globally, has seen Immune checkpoint inhibitors(ICIs) pioneer a promising therapeutic avenue. However, the response rate to ICIs remains below 30%, compounded by challenges such as resistance and immune-related adverse events (6, 7). The gut microbiota and their metabolites interact with various immune cells to promote antitumor immunity. In the tumor microenvironment (TME) (8), Bacteroides thetaiotaomicron induces M1 macrophage polarization, thereby exerting antitumor effects (9).

The microbiota has been identified as a pivotal factor influencing multiple diseases, and its relationship with HCC has emerged as a critical research focus. In this review, we focus on elucidating the mechanistic contributions of the microbiota to HCC pathogenesis. Additionally, we discuss gut microbiota-modulating strategies with therapeutic potential in HCC, alongside the impact of microbiota on HCC immunotherapy, particularly in responses to ICIs. Finally, we address the prognostic implications of microbial communities in HCC, highlighting specific microbial signatures as prognostic biomarkers.

## 2 Gut-liver axis

**Figure 1:**
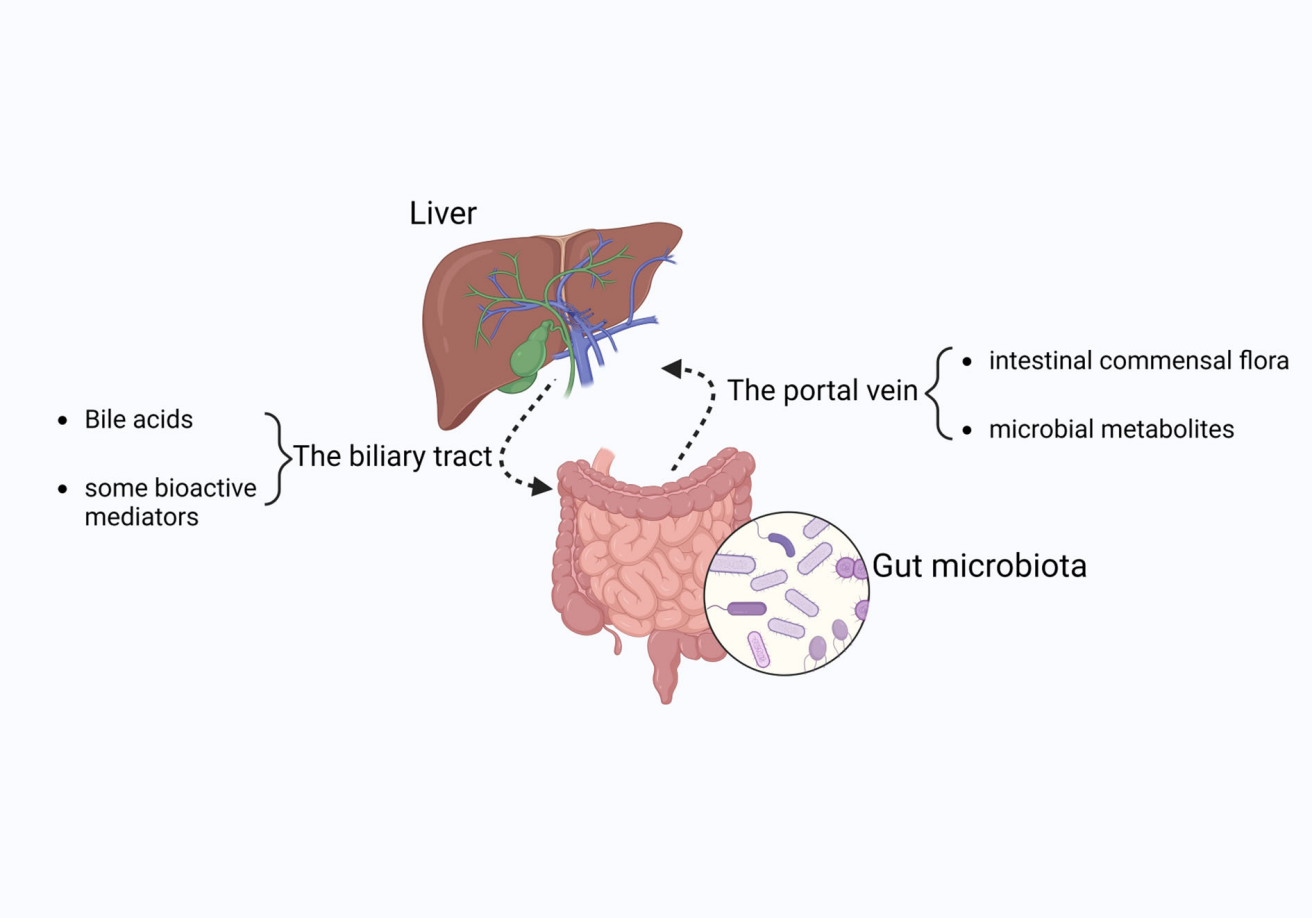
The gut-liver axis is a crucial body function. Commensal gut bacteria and microbial metabolites travel to the liver via the portal vein, while bile acids and other bioactive substances are delivered through the biliary tract. The figure was created using BioRender.com.

Anatomically and functionally, the gut and liver have a bidirectional association known as the gut-liver axis, which is primarily connected by the portal vein, the biliary tract, and the systemic circulation (10). It has been suggested that the portal vein is the most crucial component of the gut-liver axis, as it carries 70% of the blood from the gut to the liver. Therefore, the portal vein allows the entrance of intestinal commensal flora and microbial metabolites into the liver (11). Under normal conditions, the liver delivers bile acids and bioactive substances to the intestines through the bile ducts and the bloodstream (12, 13) (**Figure 1**).

In healthy individuals, the intestinal barrier protects them from intestinal commensal bacteria and allows only a few potentially pathogenic bacteria or toxic substances to enter the portal vein. However, high-fat diets, long-term alcohol consumption, viral infections, autoimmune diseases, and inflammatory bowel diseases can compromise the gut barrier integrity and increase gut permeability (14, 15). These alterations influence the gut microflora composition and cause ecological dysregulation. Gut microbiota alterations and increased intestinal permeability promote the translocation of microbe-associated molecular patterns (MAMPs) and bacterial metabolites to the liver through the portal vein. Continuous exposure of the liver to MAMPs and bacterial metabolites promotes chronic liver diseases, including hepatitis and cirrhosis, which ultimately leads to HCC. Moreover, these alterations affect both intestinal and hepatic immune functions (16). Researchers have discovered that the gut microbiota diversity in patients alters significantly during the development of HCC (17). Overall, these studies indicated that the gut-liver axis indicates the association between the gut and the liver. Are intratumoral microbiota inherently resident in the liver or gut-derived? This is a question worthy of thought.

## 3 Liver cancer-associated microbiota

This section describes the microbial composition of the gut and liver tissues of healthy individuals and then discusses the microbiota composition and abundance heterogeneity within HCC tumors of different etiological origins to provide a basis for future studies on HCC development, diagnosis, and prognosis. In all HCC samples analyzed for microbiota composition in this study, we systematically excluded individuals who had received antibiotic therapy, chemotherapy, or radiotherapy to ensure the reliability of causal associations between microbial signatures and HCC progression.

### 3.1 Composition of the liver microbiota and gut microbiota in healthy individuals

The healthy gut microbiota comprises four main phyla: Firmicutes, Bacteroidetes, Actinobacteria, and Verrucomicrobia (18). Historically, normal liver tissue was considered sterile due to the limited sensitivity of early detection technologies. However, high-throughput sequencing studies have revealed distinct microbial communities in both normal hepatic parenchyma and adjacent peritumoral tissues. Peritumoral tissue samples are predominantly colonized by the phyla Proteobacteria, Firmicutes, Actinobacteria, and Bacteroidetes (19), whereas normal liver tissues primarily harbor Patescibacteria, Proteobacteria, Bacteroidota, Firmicutes, and Actinobacteriota (20). Understanding the composition of the liver and gut microbiota in healthy individuals provides baseline data on the microbiota under normal conditions, which helps to reveal how dysbiosis may influence the onset and progression of HCC through the gut-liver axis. The gut and liver microbiota of healthy individuals exhibits relatively conserved community features at the phylum level, while the composition of lower taxonomic ranks (e.g., genus and species) demonstrates significant heterogeneity modulated by age, dietary habits, geographic location, and therapeutic interventions. The studies of the related microbiota in healthy individuals are summarized in **Table 1**.

**Table 1:** Studies on the related microbiota in healthy individuals.

Author	Studied subjective (n)	Microbiota type	Sampled related parts	Method	Enriched microbiota characteristics
Sun (19)	Healthy individuals (n *=* 30)	Liver microbiota	Paracancerous tissue samples	16srRNA	Phylum: Proteobacteria, Firmicutes, Actinobacteria Genus: *Rhodococcus*, *Azoarcus*, *Ochrobactrum*, *Klebsiella*
Huang (20)	Healthy individuals (n *=* 28)	Liver microbiota	Paracancerous tissue samples	16srRNA	Phylum: Proteobacteria, Firmicutes, Actinobacteriota Classes: Bacilli, Actinobacteria
Yan F (87)	Healthy adults (n *=* 30)	Gut microbiota	Fecal samples	16srRNA	Phylum: Bacteroidoata, Firmicutes, Proteobacteria Family: Bacteroidaceae, Enterobacteriaceae, Lachnospiraceae
Lai (88)	Healthy individuals (n *=* 17)	Gut microbiota	Fecal samples	16srRNA	Genus: *Bacteroides*, *Prevotella, Faecalibacterium*
Huo (89)	Healthy Individuals (n *=* 20)	Gut microbiota	Fecal samples	16srRNA	Phylum: Actinobacteriota Family: Lachnospiraceae, Ruminococcaceae, Bifidobacteriaceae Genus: *Blautia*, *Faecalibacterium, Bifidobacterium*
Chen (90)	Healthy individuals (n *=* 9)	Gut microbiota	Fecal samples	16srRNA	Phylum: Firmicutes Genus: *Blantia*
Liu (32)	Healthy controls (n = 33)	Gut microbiota	Fecal samples	16srRNA	Phylum: Firmicutes, Proteobacteria, Bacteroidoata Order: Bacteroidales, Clostridiales, Enterobscterriales Genus: *Bacteroides, Prevotella, Faecalibacterium*

All the content in the table is from the references in the article.

### 3.2 Microbial alterations from chronic liver disease to liver cancer

**Figure 2:**
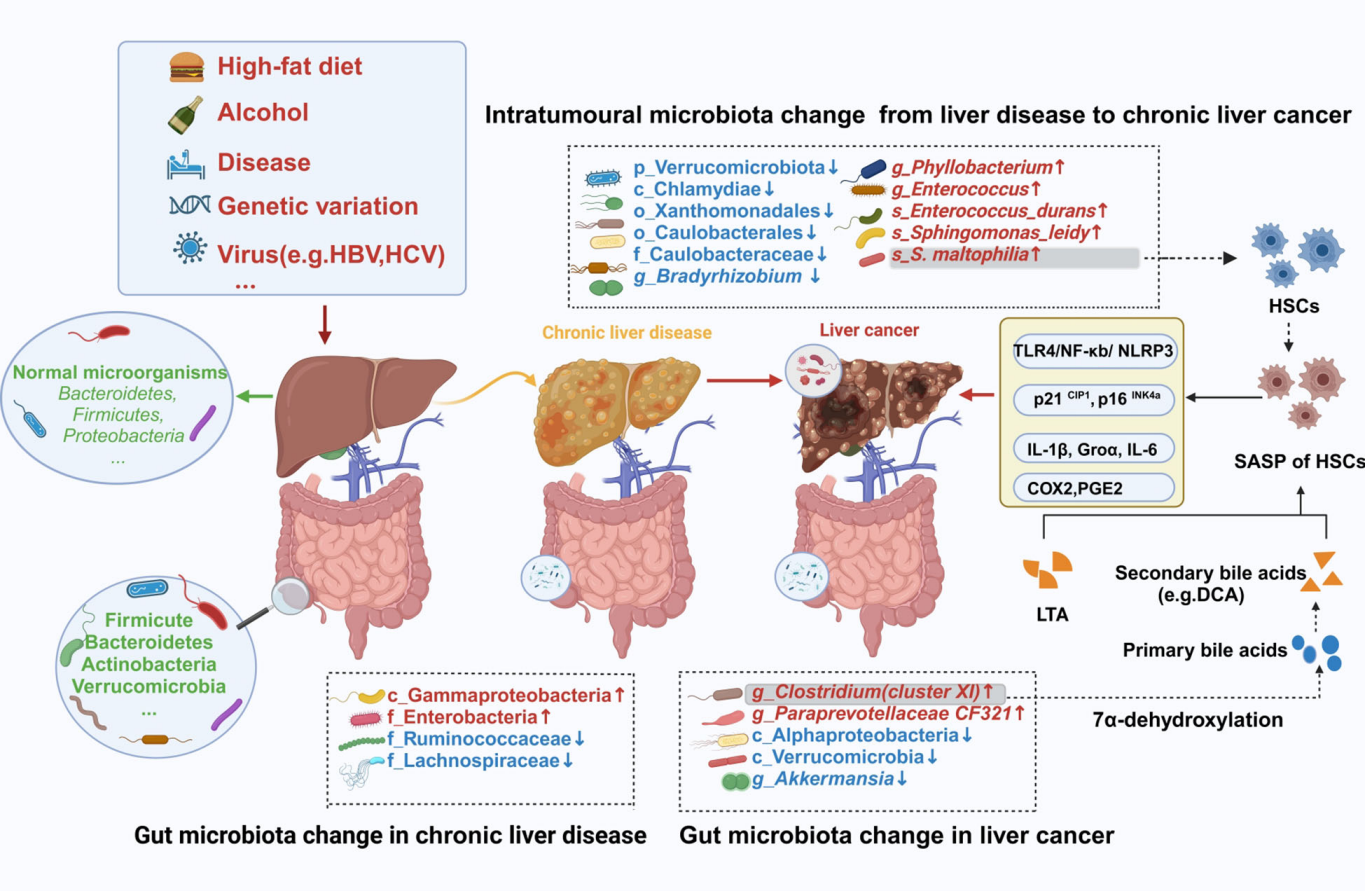
Changes in the intestinal and intratumoral microbiota during hepatocarcinogenesis. Intestinal barrier and liver damage can be caused by some external factors, including a high-fat diet, alcohol, diseases, and viruses. Furthermore, the intestinal and intratumoral microbiotas are disrupted, causing dysbiosis. Moreover, intratumoral tissues and intestinal sites comprise specific pathogenic bacteria, which exert their cancer-promoting effects through specific mechanisms. HBV, hepatitis B virus; HCV, hepatitis C virus; HSCs, hepatic stellate cells; SASP, senescence-associated secretory phenotype; LTA, lipoteichoic acid; DCA, deoxycholic acid. The figure was created using BioRender.com.

HCC has been proposed to develop from the progression of chronic liver disease into cirrhosis and then HCC. Current studies on the evolution of cirrhosis to HCC suggest the crucial role of the intrahepatic microbiota. Liver inflammation or damage caused by certain viruses can lead to chronic hepatitis, which, if left untreated, can progress to cirrhosis and eventually HCC. A study demonstrated that tumor tissues of HCC patients were enriched with the genera Stenotrophomonas, Acinetobacter, Phyllobacterium, and Enterococcus. Furthermore, a diagnostic model combining *Stenotrophomonas* and *Romboutsia* achieved an area under the curve (AUC) value of 0.78 for HCC diagnosis (21). Furthermore, they co-detected *Stenotrophomonas maltophilia (S. maltophilia)*, a species of the genus *Stenotrophomonas*, in intratumor and para-tumor liver tissues. The integrated diagnostic model, combining key microbial taxa(including *Stenotrophomonas*) from oral and fecal sources with alpha-fetoprotein (AFP), significantly improves HCC discrimination accuracy (AUC = 0.9811), outperforming standalone microbial models or AFP-based biomarkers (22). Therefore, the origin of intratumoral *Stenotrophomonas* colonization remains to be fully elucidated. Given the existence of the gut-liver axis, the gut microbiota in patients with HCC undergoes dynamic changes. Research has also shown a notable change in the gut microbiota composition of patients with HCC, both with and without cirrhosis, when compared to healthy individuals (23). Moreover, significant enrichment of the class Gammaproteobacteria and the family Enterobacteria, while reduced abundance of the families Ruminococcaceae and Lachnospiraceae, has been observed in the intestines of cirrhosis patients. Comparative analysis revealed significant gut microbial shifts in cirrhotic HCC patients: *Clostridium* and *CF231* genera were enriched, whereas *Akkermansia muciniphila(Akkermansia)* is depleted (24). Among these, the genera *CF231* and *Clostridium* could be the primary bacteria linked to the advancement of cirrhosis into HCC. Behary et al. (25) found that the intestines of nonalcoholic fatty liver disease (NAFLD)-HCC patients were enriched with five species (e.g., *Bacteroides caecimuris, Veillonella parvula, Bacteroides xylanisolvens, Ruminococcus gnavus*, and *Clostridium bolteae*). The enrichment of *Clostridium* spp. in the gut of NAFLD-HCC and cirrhosis-HCC patients suggested a potential role for these bacteria in HCC development caused by other etiologies. To elucidate this hypothesis, further studies on the gut microbiological composition of HCC patients with different etiologies, particularly focusing on the role of *Clostridium* spp, should be carried out. The collective findings of these studies confirm the presence of microbial alterations in the gut or intratumoral regions in HCC patients (**Figure 2**). Recently, the study of intestinal fungi in tumor patients has gradually become a research hotspot. Two studies investigating changes in intestinal fungi during HCC progression have found that *Candida albicans* is significantly enriched in the intestines of HCC patients (26, 27). Of these two, one of the studies further validated its carcinogenic effects via mouse experiments (27). However, the specific carcinogenic mechanisms of *Candida albicans* in HCC remain elusive.

### 3.3 Microbial differences between HBV-positive and –negative liver cancer

Chronic hepatitis mainly includes chronic hepatitis B, chronic hepatitis C, alcoholic hepatitis, non-alcoholic steatohepatitis, and autoimmune hepatitis. Chronic hepatitis B caused by HBV accounts for 56% of viral hepatitis (28). Persistent HBV infection is a global public health concern and requires hepatitis B monitoring. Untreated chronic hepatitis B may cause persistent liver damage and develop into cirrhosis and, eventually, HCC.

HBV infection significantly increases the risk of developing HCC compared to non-infected individuals. Several studies have indicated significant differences in the microbial composition between healthy people, HBV-positive HCC patients, and HBV-negative HCC patients. Furthermore, certain differences exist in the intratumoral microbial composition between HBV-associated HCC patients and non-viral HCC patients. Liu et al. (29) found that *Cutibacterium*, *Ruminococcus 2, Alcaligenes, Bacteroidales*, and *Flavonifractor* were enriched in HBV-HCC patient’s tumor tissues compared to non-HBV-HCC patients, with *Cutibacterium* being considered a biomarker for viral HCC. Another study suggested *Ruminococcus gnavus* as the tumor tissue biomarker for HBV-HCC (30). The differences in the biomarkers of these two studies might be because of the differences in study methodology, sample selection, TME, and technical analysis. Based on their microbial community structure, Li et al. (31) classified the microorganisms within liver tumors into bacterial-dominant and HBV-dominant types. They found that the bacterial-dominant type was more aggressive and had a poorer prognosis, suggesting a certain heterogeneity of intratumoral microorganisms in HCC and a possible close association with liver tumorigenesis and prognosis. The key intratumoral pathogenic bacteria responsible for the development of viral HCC require further identification. In addition, there are some variabilities in gut microbial composition; for instance, some studies have indicated increased anti-inflammatory bacteria (e.g., *Prevotella* and *Faecalibacterium*) and decreased pro-inflammatory bacteria (e.g., *Escherichia-Shigella* and *Enterococcus*) in the intestinal tracts of HBV-HCC patients. In contrast, non-viral HCC patients show a higher abundance of pro-inflammatory bacteria (e.g., *Escherichia-Shigella* and *Enterococcus*) in the gut, while *Faecalibacterium, Ruminococcus, Ruminoclostridium*, and others are less abundant (32). ROC curve analysis using a random forest classifier, based on 16 genera(including *Faecalibacterium,Prevotella,and Ruminococcus* and so on) significantly distinct between viral and non-viral HCC, achieved an AUC value of 0.94 for diagnosing viral HCC (33). These data suggest that HBV may have some effect on changes in the gut flora of HCC patients.

## 4 Liver carcinogenesis

Microbiological research indicates that viruses, along with specific intratumoral or intestinal bacteria, significantly contribute to the progression of HCC.

### 4.1 Effect of HBV infection on HCC

HBV infection has been closely associated with HCC (34), with 90% of HCC patients harboring the hepatitis B virus. In the early stages, researchers detected HBV DNA in both liver and tumor tissues of HBV-infected HCC patients (35). Therefore, we conducted an in-depth investigation into the mechanisms by which HBV contributes to HCC development, aiming to elucidate its specific role in carcinogenesis and the underlying biological processes.

#### 4.1.1 Carcinogenic mechanisms of HBV

HBV induces HCC through several key mechanisms. Initially, a key element in the progression of the majority of HCCs linked to HBV is the incorporation of HBV DNA into the genome of the host. Advanced sequencing techniques have shown that the X and C genes of HBV are primarily integrated into the host genome (36). Among these, the X gene plays a major role in promoting the expression of oncogenes (e.g., Myc, Src, and CyclinD1) and inhibiting tumor suppressor genes (e.g., P53 and Rb), which drives hepatocellular carcinogenesis (37). Secondly, HBV can regulate the expression of microRNAs through various mechanisms, facilitating its own replication (38). Additionally, HBV structural proteins, such as the HBx protein, can activate oncogene expression or silence tumor suppressor gene expression via multiple pathways, thereby promoting the development and progression of HBV-related HCC (39–41). The mechanisms of HBV action require further in-depth research and exploration.

#### 4.1.2 HBV and other microbiota

The role of HBV in the development of HCC has been thoroughly studied. In recent years, an increasing number of studies have started investigating the link between HBV-induced microbiota dysbiosis and HCC. Research based on 16S rRNA sequencing has shown that HBV infection may induce changes in the composition of the gut microbiota, thereby promoting the progression of HCC (32, 42). Intestinal microbiota dysbiosis affects liver health through the gut-liver axis, primarily by triggering chronic inflammation, bile acid metabolism disorders, oxidative stress, and immune evasion, which collectively increase the risk of HCC. These changes in microbial abundance are closely linked to the regulation of stem cell function and the immune system, thereby promoting tumor development and progression. In conclusion, the specific mechanisms underlying HBV-mediated alterations in the microbiota still require further validation through both *in vivo* and *in vitro* studies.

### 4.2 The role of specific bacteria in the development of liver cancer

Several studies have validated the close two-way communication between the gut and liver. Most previous studies have demonstrated that metabolites produced by intestinal flora enter the liver tissue via the gut-liver axis and promote liver damage, thereby causing HCC. However, in recent years, it has been found that the tumor tissues of HCC people not only have intestinal flora metabolites but also specific intestinal pathogenic bacteria (**Figure 2**). For example, Yang et al. (22) analyzed the enriched microbiota within the tumor using 46 HCC liver tumors, 42 normal paracancerous, and 11 liver hemangioma biopsy tissue samples. They showed that many bacterial genera were present in the tumor during the progression of HCC, such as *Enterococcus, Escherichia Shigella, Streptococcus*, and *S. maltophilia*, which were also present in the gut of HCC patients. *Enterococcus* and *Escherichia Shigella(E. Shigella)* are alcohol-related pro-inflammatory bacteria that can aggravate intestinal leakage and lead to dysbiosis. Furthermore, the overgrowth of *E. Shigella* can also disrupt the health balance and enter the liver via blood circulation, thereby promoting the progression of NAFLD to HCC in non-alcoholic fatty liver disease patients (43). The emerging multi-drug resistant *S. maltophilia* is a globally recognized opportunistic pathogen that poses a significant threat to cancer patients (44). It is a dominant strain in HCC patients, which can promote the progression of liver cirrhosis into HCC. The underlying mechanism of this progression primarily includes the activation of the TLR-4-mediated NF-κB signaling pathway, induction of the senescence-associated secretory phenotype (SASP) of hepatic stellate cells (HSCs), formation of NLRP3 inflammasome complex, secretion of various inflammatory factors in the liver, and progression of HCC (21). *S. maltophilia* also activated the Calcium-ROS-AMPK-mTOR-autophagy pathway by migrating from the gut to the mammary gland via the gut-mammary axis, thus causing mastitis (45). This suggests that *S. maltophilia* is crucially involved in the development of various diseases and warrants further research. Therefore, it can be inferred that *S. maltophilia* is a promising target for preventing liver cirrhosis progression to HCC. Recent studies have demonstrated that the gut pathobiont *Klebsiella pneumoniae(K. pneumoniae)*, enriched in liver tissue, interacts with TLR4 on hepatocellular carcinoma (HCC) cells via its surface protein PBP1B, activating the TLR4-mediated oncogenic signaling pathway to promote tumor progression (46). Furthermore, whether *Klebsiella pneumoniae*-derived metabolites exacerbate HCC pathogenesis remains to be experimentally validated.

Several studies have indicated that the key changes in the intestinal flora of cirrhosis patients are a decrease in beneficial bacteria and an increase in pathogenic bacteria (10, 47). *Clostridium cluster XIVa* belongs to a bacterial taxon of the genus *Clostridium*. The high-fat diet-fed mice indicated an increase in the intestinal Gram-positive strains, specifically *Clostridium cluster XI* (48). *Clostridium cluster XI* can transform primary bile acids into secondary bile acid deoxycholic acid (DCA) via 7α-dehydroxylation. Furthermore, this DCA synergistically interacts with the TLR2 agonist lipoteichoic acid (LTA) to induce an HSC senescence-associated secretory phenotype, thereby suppressing anti-tumor immunity via a prostaglandin E2-dependent mechanism and ultimately promoting the progression of HCC (49). A study on NAFLD-HCC concluded that *Clostridium bolteae* modulates the immune system by producing short-chain fatty acids (SCFAs), thereby inducing increased expression of Tregs and suppressing CD8+ T-cell activity (25). This creates an immunosuppressive environment that is conducive to tumor growth. This implies that exploring the relationships among the microbiota, its metabolite, and the immune system will be a crucial avenue for comprehending the processes underlying the development of HCC.

## 5 The role of microbial interventions in liver cancer therapy

**Figure 3:**
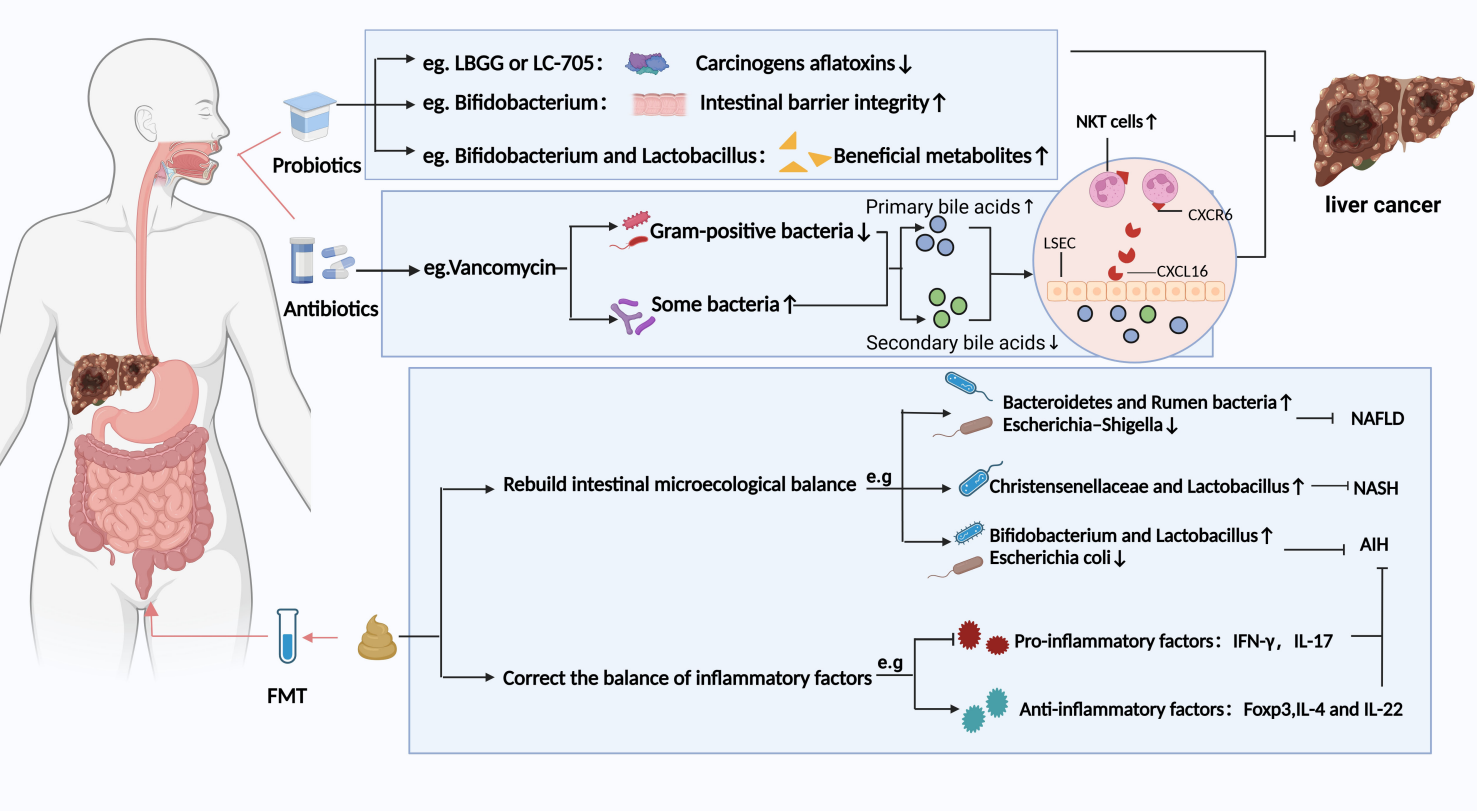
Potential strategies based on gut microbiota modulation for preventing HCC and chronic liver disease. Different strategies have different prevention and treatment mechanisms. LBGG, Lactobacillus rhamnosus GG; LC-705, Lactobacillus rhamnosus LC-705; NKT, natural killer T; LSEC, liver sinusoidal endothelial cells; FMT, fecal microbiota transplantation; NAFLD, nonalcoholic fatty liver disease; NASH, Non-alcoholic steatohepatitis; AIH, Autoimmune hepatitis. Figure created with BioRender.com.

The gut-liver axis not only modulates the progression of chronic liver disease and HCC but also serves as an indispensable target for HCC treatment. The specific mechanisms of targeting intestinal microbiota balance include regulating the composition of related metabolites and related inflammatory factors. Currently, in clinical practice, a range of adjuvant therapies targeting microorganisms have been developed as complementary approaches to standard treatment. These therapies include probiotics, fecal microbiota transplantation (FMT), antibiotic therapy, among others (**Figure 3**).

### 5.1 Probiotics, prebiotics, synbiotics and postbiotics

**Figure 4:**
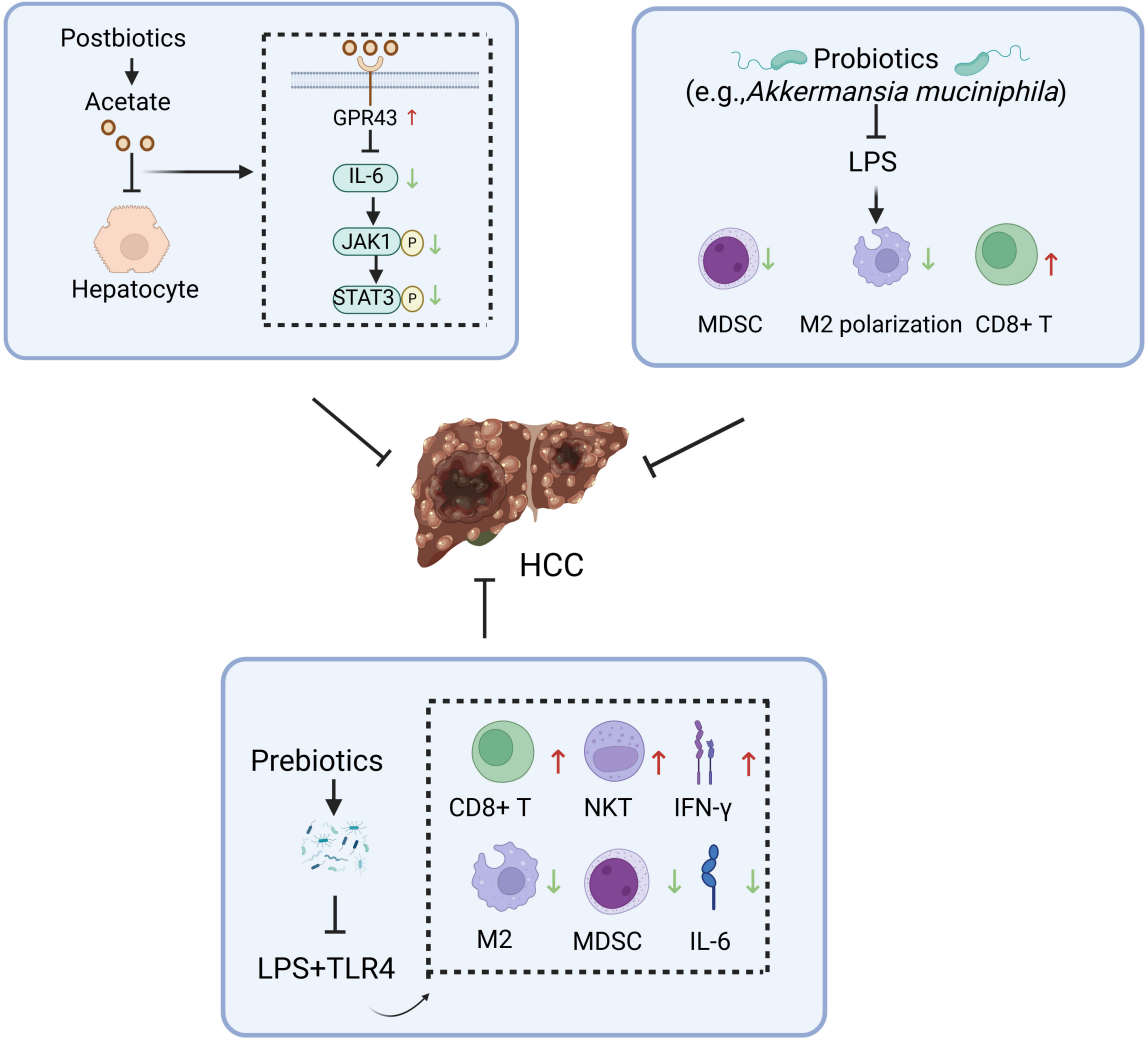
Potential mechanisms underlying the inhibitory effects of gut microbiota modulators against hepatocellular carcinoma. HCC, hepatocellular carcinoma; GPR43, G coupled-protein receptor 43; NKT, natural killer T; IFN-γ, interferon-γ; MDSC, myeloid-derived suppressor cell; LPS, lipopolysaccharide. Figure created with BioRender.com.

Probiotics, as defined by the United Nations and the World Health Organization, refer to living microorganisms that benefit the host when administered in adequate quantities (50). Recently, probiotics have been increasingly used to treat and prevent disease. For instance, they have been suggested to play a beneficial role in treating and preventing HCC through various mechanisms (51). Current research demonstrates that specific probiotics can effectively counteract the carcinogenic toxicity of aflatoxins (AFT) through adsorption or binding mechanisms. Although the underlying mechanisms remain incompletely elucidated, significant biological effects have been observed: *Lactobacillus casei GG* (LGG) and strain LC-705 exhibit high-affinity binding to AFT, markedly reducing its bioavailability and toxicity (52). Clinical evidence further confirms that LC-705 intervention lowers urinary levels of AFT metabolic biomarkers in patients, suggesting chemopreventive potential against HCC (53). Notably, an optimized probiotic consortium of *Lactobacillus casei* and *Saccharomyces cerevisiae* achieves a 98.4% AFT removal rate in contaminated dairy products (54), providing critical experimental support for probiotic-mediated food safety intervention strategies. Secondly, probiotics can regulate the ecological balance of gut flora, thus preserving the integrity of the intestinal barrier. *Bifidobacterium pseudolongum* suppresses the progression of NAFLD-HCC (55). *Lactobacillus* strains (e.g., *Levilactobacillus brevis* SR52–2 and *LeviLactobacillus delbrueckii subsp.bulgaicus* Q80) with anti-HBV activity reduce the risk of chronic viral inflammation-driven HCC by remodeling the gut microbiota ecosystem in HCC patients (56). In addition, probiotics may also inhibit the progression of HCC by producing beneficial bacterial metabolites. Probiotics enrich short-chain fatty acid (SCFA)-producing taxa and significantly elevate the biosynthesis of SCFAs within the intestinal lumen (57). SCFAs reduce the risk of malignant transformation from chronic liver disease to HCC in high-risk HBV carriers by targeting the tumor suppressor DAB2 (58). Prebiotics are substrates selectively utilized by host microorganisms, conferring health benefits. Common types of prebiotics primarily encompass oligosaccharides (e.g.,fructooligosaccharides, galactooligosaccharides, inulin), microalgae (e.g., *Spirulina*, *Arthrospira*), and protein hydrolysates (e.g., lactoferrin). Wang et al. (59) have demonstrated that an oral dextran-carboxymethyl deoxycholate conjugate nanotherapeutic agent exerts therapeutic effects against HCC by modulating the gut microbiota and remodeling the TME through coordinated mechanisms (**Figure 4**). Synbiotics are microecological formulations combining probiotics and prebiotics, which may confer therapeutic benefits to HCC patients through their synergistic actions (60). Postbiotics, an emerging concept in microbiome research, refer to complexes consisting of microorganisms inactivated through specific processes, combined with their metabolites and cellular components. Probiotics and postbiotics complement each other and are expected to improve the efficacy and survival of ICIs in patients with HCC (60).

### 5.2 Fecal microbiota transplantation

The FMT is a process of introducing fecal flora from a healthy individual into a patient’s gut to modify the patient’s gut flora homeostasis (60). A study on FMT for patients with recurrent *Clostridium difficile* infections demonstrated that FMT can significantly alter the composition of the gut microbiota (61). Similarly, FMT treatment also has a significant impact on the composition of the gut microbiota in patients with chronic liver disease. For example, FMT has been observed to improve NAFLD by restoring the gut microbiota balance, especially in lean NAFLD patients. This is evidenced by a reduction in average fat levels to normal and the alleviation of gastrointestinal symptoms such as chronic diarrhea and constipation (62). This reconstitution was characterized by an increase in the butyric acid-producing *Bacteroidetes* and *Rumen* bacteria and a decrease in the intestinal opportunistic pathogen *Escherichia-Shigella*. A clinical trial (NCT03420482) suggested that FMT may improve the quality of life in patients with ALD (63). In another investigation, the steatohepatitis mice induced by a high-fat diet were treated with FMT for eight weeks. These mice showed an increased abundance of the beneficial gut microbiota *Christensenellaceae* and *Lactobacillus*, which corrected intestinal flora disorders and thus attenuated steatohepatitis (64). Liang et al. (65) analyzed the effect of FMT on the progression of autoimmune hepatitis in mice. They found that FMT reestablished the balance of gut microbiota in mice (e.g., increase in *Bifidobacterium* and *Lactobacillus, Escherichia coli* depletion) to mitigate autoimmune hepatitis. However, whole genome sequencing is required to investigate the microbiota and the interactions with their metabolites in FMT-treated HCC patients. How FMT cures HCC by correcting the intestinal microecological balance needs further clinical studies.

### 5.3 Antibiotics

Antibiotics can inhibit chronic liver disease from progressing to HCC by targeting gut flora. The antibiotics act via two main routes: 1) reduce the abundance of pathogenic bacteria with a strong translocation ability and 2) mediate or reduce the bacterial metabolites. The vancomycin dselectivelyepletes Gram-positive bacterial communities, driving the biotransformation of primary bile acids (e.g., cholic acid) into secondary bile acids (e.g., deoxycholic acid). This metabolic reprogramming upregulates hepatic recruitment and activation of CXCR6^+^ natural killer T (NKT) cells via bile acid receptor signaling, ultimately preventing HCC (66–68) (**Figure 3**). In oncological clinical management, prophylactic antibiotic administration is routinely integrated into perioperative or systemic therapeutic regimens for cancer patients to mitigate the risk of opportunistic pathogen infections under immunocompromised conditions. However, Antibiotic treatment also has some negative effects. Antibiotic treatment significantly disrupts gut microbiota homeostasis and impairs the clinical response rates and survival benefits of ICIs patients (69, 70). This implies that future research should emphasize rational antibiotic use to strike a balance between therapeutic efficacy and microbiota preservation.

## 6 Microbiota is associated with the prognosis of liver cancer

Aggressive malignant tumors like HCC have a high recurrence rate and a dismal prognosis. Patients with such tumors have significantly different clinical outcomes and treatment effects at the same or different clinical stages. Therefore, screening can predict the prognosis of HCC biomarkers, which is necessary for the complete cure of HCC. Research has indicated that the microbiota present within tumors in the TME is closely linked to postoperative outcomes and impacts on individuals suffering from HCC. Qu et al. compared the postoperative intratumoral microbiota in long-term survivors (LTS) and short-term survivors (STSs) (71). The findings indicated variations in the quantities of bacterial families and genera observed postoperatively between LTS and STS. Furthermore, it was observed that the tumor tissues of LTS were enriched with *Pseudomonas*, *Thermomonas*, Paraprevotellaceae, and other bacteria at the family or genus level. Whereas tumor tissue of STS was enriched with Enhydrobater, Lachnospiraceae, and Deltaprotepbacteria. The bacterium with the most significant difference between the two groups was Pseudomonas, suggesting that it might have crucial effects on the outcome of primary HCC patients. Recently, Song et al. (72) constructed a microbiota-associated scoring model based on 27 intratumoral microorganisms to predict the length of overall HCC patient survival. They found that enriched microbes, such as *Akkermansia* and *Methylobacterium*, predict better prognosis in HCC patients (73). Another study indicated a correlation between intratumoral *Methylobacterium* and unfavorable prognosis among gastric cancer patients (74). These differing findings suggest that organisms could play unique roles in different anatomical locations or pathological situations. Further research is warranted to investigate the impact of TME-critical microbes on the prognosis of HCC. It has been inferred that a prognostic biomarker for HCC can be derived from the gut microbiota. Ni J et al. (75) investigated the correlation between the extent of gut microbiota dysbiosis and HCC progression in primary HCC patients to identify potential microbial markers with prognostic value. They indicated a positive correlation between the stage of primary HCC and the extent of dysbiosis in the intestinal flora. Compared to the healthy people, stage II and III primary HCC patients had a relatively increased abundance of Proteobacteria at the phylum level. Moreover, the intestines of stage I HCC patients were enriched with many bacterial genera, including *Actinomyces*, *Atopobium*, *Paraprevotella*, *Veillonella*, and other unidentified genera.

By integrating the analyses of both microbiotas as combined prognostic markers, researchers may be able to significantly improve the accuracy of prognosis assessments for HCC patients, leading to better-informed clinical decision-making and tailored treatment strategies.

## 7 Influence of the gut microbiota on liver cancer immunotherapy

The gut microbiota critically regulates the efficacy of HCC immunotherapy through two aspects: (1) the gut microbiota affects immune cell, and (2) metabolites of the gut microbiota regulate antitumor immunotherapy.

### 7.1 Direct stimulation of immune cells

Certain gut microbiota can trigger specific antitumor immune cell. Early studies have begun to elucidate the cascade regulatory mechanisms linking the microbiota and immune cells. For instance, *Bifidobacterium* enhances the antigen-presenting capacity of dendritic cells (DCs), thereby driving the activation and recruitment of CD8+ T cells within the TME (76). Subsequent studies have confirmed that *Lachnospiraceae bacterium* GAM79 enhances anti-tumor immune responses by remodeling the tumor immune microenvironment, thereby significantly extending progression-free survival (PFS) and overall survival (OS) in HCC patients treated with programmed death-1 (PD-1) inhibitors (77). In recent years, Akkermansia, a representative gut commensal bacterium, has garnered significant attention in cancer immunotherapy research. HCC patients responsive to anti-PD-1 immunotherapy exhibited gut microbiota enrichment of *Akkermansia (* 78). This phenomenon may be attributed to *Akkermansia*’s suppression of monocytic myeloid-derived suppressor cells (m-MDSCs) and M2 macrophages, thereby remodeling the immunosuppressive tumor microenvironment in HCC (79). *Akkermansia* may enhance the efficacy of anti-PD-1 immunotherapy.

### 7.2 Metabolites regulate immunotherapy

Small-molecule metabolites directly synthesized or co-metabolized by the gut microbiota through host-microbiota interactions modulate local and systemic anti-tumor immune responses via local diffusion and gut-liver axis signaling, thereby enhancing the efficacy of ICIs. The gut microbiota produces three major classes of key immunomodulatory metabolites. The first key immunomodulatory metabolite is inosine, biosynthesized as a purine-derived compound by *Lactobacillus johnsonii* (80), *Bifidobacterium muciniphila* (81), and *Bifidobacterium infantis* (82), with functional significance in adenosine receptor-mediated immunoregulation. Inosine induces T helper 1 (Th1) cell activation in a microenvironment-dependent manner via the T cell-specific adenosine A2A receptor (A2AR) signaling pathway, while the inosine-A2AR axis mediates the therapeutic potentiation of immune checkpoint blockade (ICB) (83). The gut microbiota produces two key metabolites: bile acids and SCFAs. Studies demonstrate a distinct enrichment of secondary bile acid metabolites and SCFAs in fecal samples from HCC patients who responded to immune checkpoint inhibitor (ICI) therapy and achieved significant survival benefits (76, 84). Secondary bile acids, produced through the conversion of primary bile acids by gut microbiota, are subsequently carried back to the liver through enterohepatic circulation. These metabolites upregulate hepatic CXCR6^+^ natural killer T (NKT) cells, thereby suppressing and eliminating HCC cells while enhancing the therapeutic efficacy of ICIs (67). SCFAs include acetate, propionate, and butyrate. Acetate dually drives macrophage M1 polarization through ACC1-dependent fatty acid synthesis and histone acetylation, thereby synergistically activating the anti-tumor function of CD8^+^ T cells via metabolic support and cytokine coordination (85). In addition, butyrate facilitates the differentiation of activated CD8+ T cells into memory T cells (86).

Investigating the potential of specific microbial biomarkers and their metabolites to predict immunotherapy responses may significantly enhance HCC immunotherapy outcomes.

## 8 Summary and prospects

In conclusion, HCC is associated with unique microbiota characteristics. In summary, microbial dysbiosis is intricately linked to the development and progression of HCC. However, gut and hepatic microbiota profiles demonstrate significant heterogeneity across study cohorts. This variability may stem from diverse environmental and lifestyle factors—including geographic variations, dietary patterns—as well as methodological discrepancies in sampling and sequencing methods. Furthermore, given the challenges in obtaining healthy liver specimens, non-invasive sampling techniques (e.g., liquid biopsy) could be strategically employed to assess microbiota composition. Thus, despite a substantial body of literature on this topic, defining a universally consistent core gut and intratumoral microbial signature in HCC remains challenging. Furthermore, while this review has identified specific gut and intratumoral pathogens as key drivers of HCC, future research can investigate the roles of fungi and extraintestinal microbiota (e.g., oral, blood) to enable a multifaceted analysis of HCC pathogenesis. Emerging evidence underscores the paramount research significance of microbiota-derived metabolites over direct microbial effects. From a translational medicine perspective, these metabolites not only hold promise as novel diagnostic biomarkers but may also serve as actionable targets for modulating therapeutic responses. Critically, their metabolic profiles inherently arise from synergistic interactions within multispecies consortia, representing functional readouts of complex microbial ecosystems. Thus, metabolome-centric investigations could strategically circumvent challenges posed by inter-cohort microbiota variability in compositional analyses. This review summarizes the microbiota dynamics influencing each stage of HCC development, elucidating their underlying mechanisms and establishing a conceptual framework for future research on precision microbiota-targeted interventions.
